# Traitement et évolution clinique de la myasthénie auto-immune au Burkina Faso

**DOI:** 10.48327/mtsi.v5i1.2025.646

**Published:** 2025-02-21

**Authors:** LOMPO Djingri Labodi, Adeline Julie Marie KYELEM, Alassane ZOUNGRANA, M. Fabienne Yabtouta KERE, Melody Zeinab GNAMPA, Hervé NACOULMA, Christian NAPON, Athanase MILLOGO

**Affiliations:** 1 Université Joseph Ki-Zerbo, Unité de formation et de recherche en sciences de la santé, Département de neurologie, Ouagadougou, Burkina Faso; 2 Centre hospitalier universitaire Tengandogo, Service de neurologie, Ouagadougou, Burkina Faso; 3 Centre hospitalier universitaire Yalgado Ouédraogo, Service de neurologie, Ouagadougou, Burkina Faso; 4 Centre hospitalier universitaire Bogodogo, Service de neurologie, Ouagadougou, Burkina Faso

**Keywords:** Myasthénie auto-immune, Ac anti-RACh, Ac anti-MuSK, Hyperplasie thymique, Thymome, Traitement, Délai diagnostique, Anticholinestérasiques, Corticoïdes, Thymectomie, Burkina Faso, Afrique subsaharienne, Autoimmune myasthenia, Anti-RACh antibodies, Anti-MuSK antibodies, Thymic hyperplasia, Thymoma, Treatment, Diagnostic delay, Anticholinesterase agents, Corticoids, Thymectomy, Burkina Faso, Sub-Saharan Africa

## Abstract

**Introduction:**

En Afrique subsaharienne, la myasthénie auto-immune (MAI) est encore peu connue et sous diagnostiquée (retard diagnostique, faible disponibilité et faible accessibilité des moyens diagnostiques et thérapeutiques d'efficacité prouvée), d'où un pronostic fonctionnel péjoratif et une mortalité élevée. La présente étude a pour but d’évaluer les modalités thérapeutiques et évolutives cliniques de la MAI au Burkina Faso.

**Patients et méthodes:**

Il s'agit d'une étude longitudinale et multicentrique, réalisée de mars 2015 à avril 2023. Elle a concerné les patients qui présentaient des signes cliniques évocateurs de myasthénie, associés à la présence dans le sérum d'anticorps (Ac) anti-récepteurs de l'acétylcholine (anti-RACh) et/ou d'Ac anti-Muscles Specific Kinase (anti-MuSK), et/ou à la présence d'un décrément >10 % à l’électroneuromyographie, et/ou avec un test thérapeutique positif aux anticholinesthérasiques oraux. Les données sur les modalités du traitement et les données évolutives cliniques ont été analysées grâce au logiciel Epi InfoTM 7.2.5.0. Une analyse bivariée avec calcul de la p-value (<0,05) a permis d'identifier les facteurs associés à l’évolution clinique défavorable.

**Résultats:**

En tout, 40 patients atteints de MAI ont été colligés avec une prédominance féminine (60 %). La médiane d’âge de début était de 25 ans (IQ=7). Les délais médians de consultation neurologique et diagnostique étaient respectivement de 21 mois (IQ=12) et 22 mois (IQ=12). La maladie affectait des adultes jeunes dans 85 % des cas, et présentait une forme généralisée pour 35 cas. Respectivement 22 patients et 4 patients parmi 33 avaient des Ac anti-RACh et des Ac anti-MuSK. Une hyperplasie thymique et un thymome ont été trouvés chez 22 patients et 6 patients sur 38 à la TDM thoracique. Tous les patients ont reçu un traitement symptomatique par anticholinestérasiques oraux et 36 un traitement de fond par corticoïdes et/ou par immunosuppresseur (azathioprine). Quatre patients sur 9 ont eu une cure d'immunoglobulines intraveineuses (IgIV) ou des échanges plasmatiques (EP) pour crises myasthéniques. Une thymectomie a été réalisée chez 16 patients sur 40. À l'issue d'un suivi ambulatoire médian de 53 mois (IQ=16), sur les 40 patients retenus dans l’étude, 6 (soit 15 %) sont décédés, 14 (35 %) étaient en rémission clinique stable, et 17 (43 %) avaient une amélioration clinique partielle.

**Conclusion:**

Au Burkina Faso, la MAI souffre d'un retard diagnostique. La quasi-totalité des patients suivis pour MAI bénéficie d'un traitement par anticholinestérasiques et corticoïdes seuls, ou associés à l'azathioprine. Les IgIV, les EP et la thymectomie restent d'accessibilité limitée. La mortalité touche près d'un patient sur 6, et la rémission clinique stable ne concerne qu'environ un tiers des patients. Une amélioration du pronostic nécessite de rendre disponibles et accessibles les moyens diagnostiques et les traitements d'efficacité prouvée, tels la thymectomie, les immunosuppresseurs, les IgIV et les EP.

## Abstract (en)

**Introduction:**

In sub-Saharan Africa, autoimmune myasthenia gravis (AMG) is still poorly known and underdiagnosed (delayed diagnosis, poor availability and accessibility of proven effective diagnostic and therapeutic tools), resulting in a poor functional prognosis and high mortality. The aim of the present study was to evaluate the therapeutic and clinical course of AMG in Burkina Faso.

**Patients and Methods:**

This was a longitudinal, multicenter study conducted from March 2015 to April 2023. It included patients with clinical signs suggestive of myasthenia associated with the presence of serum anti-acetylcholine receptor (anti-RACh) antibodies and/or anti-muscle specific kinase (anti-MuSK) antibodies, and/or with the presence of a decrease >10% on electroneuromyography, and/or with a positive therapeutic test to oral anticholinesterase drugs. Data on treatment modalities and clinical evolution were analyzed using Epi InfoTM 7.2.5.0 software. Bivariate analysis with p-value calculation (<0.05) was used to identify factors associated with adverse clinical outcome.

**Results:**

A total of 40 patients with AMG were included, with a female predominance (60%). The median age of onset was 25 years (IQ=7). The median time to neurological consultation and diagnosis was 21 months (IQ=12) and 22 months (IQ=12), respectively. The disease affected young adults in 85% of cases and was generalized in 35 cases. Anti-RACh and anti-MuSK antibodies were present in 22 and 4 of 33 patients, respectively. Thymic hyperplasia and thymoma were found on chest CT in 22 and 6 of 38 patients, respectively. All patients received symptomatic treatment with oral anticholinesterase agents and 36 patients received background treatment with corticosteroids and/or immunosuppressants (azathioprine). Four of 9 patients received a course of intravenous immunoglobulin (IVIG) or plasma exchange (PE) for myasthenic crises. Thymectomy was performed in 16 of the 40 patients. At the end of a median outpatient follow-up of 53 months (IQ=16), of the 40 patients included in the study, 6 (15%) had died, 14 (35%) were in stable clinical remission, and 17 (43%) had partial clinical improvement.

**Conclusion:**

AMG suffers from delayed diagnosis in Burkina Faso. Almost all patients treated for AMG receive anticholinesterase and corticosteroid therapy alone or in combination with azathioprine. Access to IVIG, PE and thymectomy remains limited. Mortality occurs in nearly one in six patients, and stable clinical remission affects only about one third of patients. To improve the prognosis, we need to make available and accessible diagnostic tools and treatments of proven efficacy, such as thymectomy, immunosuppressants, IVIG and PE.

## Introduction

La myasthénie *(myasthenia gravis*, MAI) est une affection auto-immune de la jonction neuromusculaire caractérisée par une fatigabilité et une faiblesse musculaires apparaissant ou s'aggravant à l'effort et diminuant ou cédant au repos. Elle est due à la présence d'auto-anticorps (auto-Ac) spécifiques dirigés contre les protéines de la membrane postsynaptique de la jonction neuromusculaire, principalement anticorps anti-récepteurs nicotiniques de l'acétylcholine (Ac anti-RACh) et Ac anti-Muscles spécifique kinase (Ac anti-MuSK) [6, 14, 17]. Ses présentations cliniques et paracliniques sont très hétérogènes, variables selon les territoires musculaires atteints, l’âge d'apparition des symptômes, les types d'auto-Ac ou les éventuelles anomalies thymiques associées. Les premières manifestations sont majoritairement oculaires avec un ptosis et une diplopie dans plus de 67 % des cas [15, 17, 39, 48]. La maladie évolue pour atteindre d'autres territoires notamment les muscles respiratoires, de la déglutition et des membres dans plus de 80 % des cas, dont la majoration rapide signe la crise myasthénique

[1, 3]. L'atteinte des muscles respiratoires en fait toute sa gravité. La prise en charge précoce et multidisciplinaire grâce aux moyens thérapeutiques actuels, notamment la corticothérapie, l'immunothérapie et la thymectomie d'une part, les échanges plasmatiques (EP), les immunoglobulines IV (IglV) et le rituximab d'autre part, a considérablement réduit sa mortalité et amélioré le pronostic fonctionnel et la qualité de vie des patients [15, 17, 39, 48]. En Afrique subsaharienne (ASS), la MAI est peu connue, sous-diagnostiquée, marquée par un retard diagnostique et de faibles disponibilité et accessibilité des moyens diagnostiques et thérapeutiques d'efficacité prouvée, d'où un pronostic fonctionnel péjoratif et une mortalité encore élevée [7, 23]. L'objectif de la présente étude est de décrire les modalités thérapeutiques et d’évaluer l’évolution clinique de la MAI au Burkina Faso, à travers une étude longitudinale descriptive et analytique.

## Patients et méthodes

Il s'est agi d'une étude longitudinale, descriptive et multicentrique en milieu hospitalier, ayant concerné l'ensemble des structures de santé privées ou publiques des 13 régions sanitaires du Burkina Faso. L’étude a couvert une période de 5 ans et 6 mois (mars 2015 à septembre 2019). Avant qu'elle ne débute, une correspondance officielle avait été transmise à tous les médecins inscrits à l’Ordre des médecins du Burkina Faso, exerçant dans les différentes structures sanitaires médicales publiques (46 centres médicaux, 13 centres hospitaliers régionaux, 5 centres hospitaliers universitaires) et dans les structures privées (350 cliniques et cabinets médicaux privés). Cette correspondance avait également été adressée aux différents responsables administratifs desdites structures sanitaires (13 directeurs régionaux de la Santé, 70 médecins chefs de district sanitaire), afin qu'ils nous communiquent tout cas suspect de myasthénie (patient présentant une symptomatologie clinique évocatrice de myasthénie) ou tout cas de myasthénie déjà confirmée. Sur un total de 368 structures de santé comptabilisées au Burkina Faso, nous en avons sollicité 218 (7 hôpitaux, 11 centres médicaux urbains et 200 structures de santé privées), parmi lesquelles 199 ont collaboré et 36 nous ont référé des cas suspects. Les cas confirmés provenaient de quatre hôpitaux, deux centres médicaux urbains et trois cliniques privées. Ainsi, les patients qui nous ont été présentés ont été régulièrement reçus en consultation externe de neurologie dans les trois principaux CHU de la ville de Ouagadougou : le CHU Tingandogo (CHU-T), le CHU Yalgado Ouédraogo et le CHU de Bogodogo. Les patients qui ne pouvaient faire le déplacement ont été vus dans leurs structures sanitaires de rattachement, après déplacement express de l’équipe des neurologues investigateurs.

Ont été inclus dans notre étude tous les patients qui avaient une symptomatologie clinique évocatrice de myasthénie, associée à au moins un des critères suivants :

- présence dans le sérum d’Ac anti-RACh à un taux > 0,4 nmol/ml (négatif ou douteux si ≤ 0,4 nmol/ml) et/ou d’Ac anti-MuSK;
- présence d'un décrément d'amplitude du potentiel d'action musculaire significatif (> 10 %) entre la 1^re^ et la 5^e^ stimulation, au minimum sur 2 couples nerf-muscle lors des stimulations répétitives à 3 cycles/seconde en électroneuromyographie de stimulation nerveuse, après arrêt des anticholinestérasiques la veille de l'examen;
- test thérapeutique par les anticholinesthé-rasiques oraux [pyridostigmine bromide, comprimés 60 mg (Mestinon^®^) ou ambeno-nium chloride, comprimés 10 mg (Mytelase^®^)] révélant un bénéfice fonctionnel net dans la vie quotidienne du patient sur une durée d'au moins 14 jours.

En tout, 78 cas suspects de myasthénie ont bénéficié d'un test thérapeutique, principalement au pyridostigmine bromide comprimés 60 mg *per os* toutes les 4 heures du lever au coucher pendant au moins 14 jours. Au total, 96 patients nous ont été référés pour suspicion de myasthénie.

N'ont pas été inclus dans l’étude les patients dont la symptomatologie clinique était évocatrice de myasthénie mais qui n’était pas confirmée par les examens paracliniques et/ou les patients ne répondant pas au test thérapeutique par anticho-linestérasique oral.

Les dosages des Ac anti-RACh et anti-MuSK n’étant pas réalisables au Burkina Faso, tous les prélèvements sériques en vue de leurs dosages ont été pratiqués puis conditionnés par une équipe de biologistes expérimentés, au sein d'un seul laboratoire de référence à Ouagadougou (Laboratoire Bio 2000) disposant d'une convention de coopération avec un laboratoire de référence en France. Les prélèvements ont ainsi été envoyés vers le laboratoire de biologie médicale CERBA, où ils ont été analysés selon la méthode de dosage radio-immunologique pour les Ac anti-RACh et la méthode d'enzymo-immunologie par test ELISA pour les Ac anti-MuSK.

Les électroneuromyogrammes (ENMG) ont été réalisés dans les laboratoires de neurophysiologie clinique des CHU de Tengandogo et de Bogodogo sur le même type d'appareils de marque Neurosoft^®^. Les couples nerf-muscle suivants ont été explorés de façon bilatérale pour chaque examen : ulnaire-abductor *digiti minimi*, médian-abductor *pollicis brevis*, axillaire-deltoïde, facial-*orbicularis oculi.*

Lors de l'admission, chaque patient colligé a été soumis à une anamnèse retraçant l'histoire de la maladie, le parcours diagnostique, les antécédents et comorbidités associées, les habitudes et mode de vie. Un examen clinique a ensuite été fait, comprenant notamment une évaluation de l’état général, des constantes, de la fatigabilité musculaire par le score musculaire moteur (SMM) initial, et une évaluation initiale de la sévérité clinique par la classification selon les stades de la *Myasthenia Gravis Foundation of America* (MGFA). De même, lors de l'admission, chaque patient a été soumis à un bilan paraclinique comprenant un ENMG de stimulation nerveuse répétitive, un dosage sérique des Ac anti-RACh et des Ac anti-MuSK, un dosage des hormones thyroïdiennes, un bilan d'auto-immunité sérique, un scanner thoracique. Cependant, en raison des coûts, seuls certains patients ont pu réaliser ces différents examens, faute de moyens financiers. Par la suite, les patients ont d'abord été mis sous traitement symptomatique anticholinestérasique. Selon l’évolution clinique, ils ont reçu dans un deuxième temps un traitement par corticoïdes *per os* ou par azathioprine. En cas d'efficacité insuffisante, la corticothérapie et l'azathioprine pouvaient être associées. Une thymectomie avec examen anatomopathologique de la pièce d'exérèse a été proposée à certains profils de patients (MAI jeunes, séropositivité à Ac anti-RACh, avec hyperplasie thymique ou MAI associée à un thymome). Certains en ont bénéficié, ayant les moyens financiers suffisants. Les autres thérapeutiques de la MAI n’étaient pas encore disponibles dans notre contexte.

Après inclusion dans l’étude, chaque patient a bénéficié d'un suivi longitudinal au long cours, avec une évaluation clinique neurologique tous les 3 mois pendant 12 mois, puis tous les 6 mois. La survenue, durant ce suivi, de poussées myas-théniques ou de crises myasthéniques avec leurs contextes de survenue, l'apparition d'effets indésirables liés aux thérapeutiques, ainsi que tout autre évènement intercurrent, ont été régulièrement notifiés et pris en charge le cas échéant. Le SMM et l’évolution clinique selon les stades de la MGFA ont été également régulièrement enregistrés.

À la fin de l’étude, les patients ont été subdivisés en survivants et en décédés et les survivants ont été classés selon le pronostic fonctionnel.

Les variables d’étude prises en compte étaient :

- sociodémographiques : âge, sexe, délai de consultation en neurologie;
- cliniques : modes d'installation (aiguë/subaiguë/progressive), délai diagnostique, SMM initial, stades MGFA;
- paracliniques : dosage des Ac anti-RACh et anti-MuSK, TDM thoracique, ENMG, examen anatomopathologique des pièces d'exérèse;
- thérapeutiques : délai de début du traitement, molécules utilisées, effets secondaires, thymectomies réalisées…;

évolutives : nombre de poussées, nombre de crises myasthéniques, contextes favorisants; évolution des indices de progression de SMM; évolution de la sévérité clinique selon les stades MGFA; mortalité; devenir clinique des survivants.

L'analyse de ces données a été faite grâce au logiciel Epi-Info 7.2.5.0. Ces données ont été présentées sous forme de pourcentage pour les variables qualitatives et sous forme de moyenne et pourcentage pour les variables quantitatives. Selon les cas, les tests T de Student et le test de Khi-2 ont été utilisés. Une analyse bivariée avec calcul de la p-value (<0,05) a permis d'identifier les facteurs associés à l’évolution clinique défavorable.

Une évolution défavorable a été définie par la constatation lors de l'examen en fin de suivi d'un état clinique stationnaire ou en aggravation, ou de la survenue d'un décès.

## Définitions opérationnelles

Une crise myasthénique a été définie comme une poussée de myasthénie impliquant les muscles respiratoires ou bulbaires, responsables d'une détresse respiratoire, nécessitant une ventilation mécanique et imposant une prise en charge immédiate en unité de soins intensifs.

Une crise cholinergique a été définie par l'association de signes muscariniques (diarrhée, hypersalivation, hypersécrétion bronchique, sueurs) et de signes nicotiniques (fasciculations, crampes, signe de Chvostek), chez un patient en surconsommation de traitements anti-cholinestérasiques. Le SMM coté de 0 à 100 points a permis d’évaluer la fatigabilité musculaire par la mesure de la force musculaire des différentes parties du corps, lors de l'examen clinique initial et, ultérieurement, lors des consultations de suivi.

Lors de l'examen clinique initial, les patients ont été subdivisés en cinq sous-groupes de gravité clinique croissante selon la classification MGFA [16, 19]. La classification des patients selon la « MGFA statut post-interventionnel » adaptée à notre contexte, a été utilisée pour l’évaluation du devenir en fin de suivi. Cette évaluation a permis d'identifier cinq modalités évolutives possibles en fin d’étude, exclusives les unes des autres :

- la rémission complète et stable ou la rémission pharmacologique : disparition complète des signes et symptômes de MAI depuis au moins un an, avec ou sans poursuite des médicaments anti myasthéniques;
- l'amélioration clinique : diminution substantielle des manifestations notées avant traitement. N'a été pris en compte que le passage d'au moins une classe MGFA à une classe moins élevée;
- l’état clinique stationnaire ou inchangé : pas de changement substantiel dans les manifestations présentes avant le traitement;
- l'aggravation clinique : augmentation substantielle des manifestations notées avant traitement, apparition de poussées ou de crises myasthéniques malgré le traitement, ou encore passage d'au moins une classe MGFA moins sévère à une classe plus sévère;
- le décès.

L’étude a été autorisée par le Comité national d’éthique du Burkina Faso. La collecte des données a été effectuée avec l'autorisation des administrations des différentes structures sanitaires. Les fiches de collecte ont été remplies sur place après avoir obtenu le consentement des patients ou de leurs tuteurs légaux. Les données recueillies sont restées confidentielles.

## Résultats

**Figure 1:**
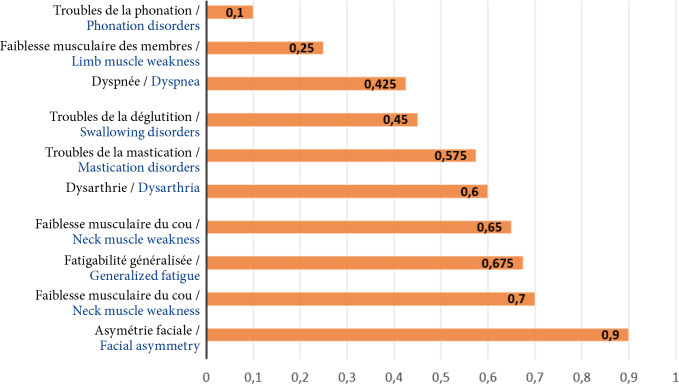
Répartition des patients selon les présentations cliniques révélatrices de la myasthénie auto-immune chez les 40 patients

Durant la période d’étude, nous avons colligé 40 patients, dont 24 femmes (60 %) et 16 hommes (40 %), soit un sexe-ratio de 1,5. La médiane d’âge de début était de 25 ans (IQ=7, extrêmes 4 ans et 71 ans). Étaient dénombrés 5 cas de MAI infanto-juvénile (13 %), 32 cas de MAI de l'adulte jeune (80 %) et 3 cas de MAI du sujet âgé (7 %). Le délai médian de consultation auprès d'un neurologue était 21 mois (IQ=12, extrêmes de 1 mois et de 18 ans). Le délai médian de diagnostic était de 22 mois (IQ=12, extrêmes 1 mois et 217 mois). La présentation clinique révélatrice était dominée par les troubles oculomoteurs ou visuels, les troubles de la phonation, la faiblesse musculaire des membres et la dyspnée par atteinte des muscles respiratoires, respectivement chez 36 patients (90 %), 28 patients (70 %), 27 patients (68 %) et 26 patients (65 %) (Fig. 1).

**Figure 2:**
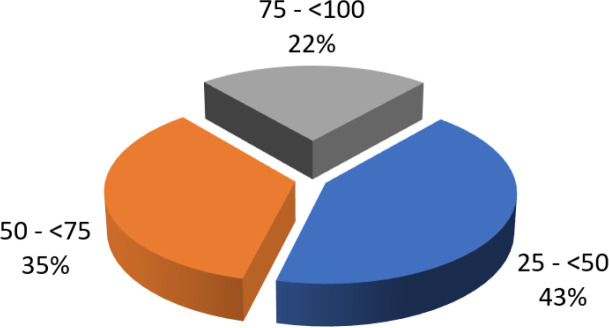
Répartition des patients selon l'importance de la fatigabilité musculaire initiale par le score SMM

Lors de l'examen clinique initial, l’évaluation de la fatigabilité musculaire a été réalisée par un SMM médian de 57 points/100 (IQ=35, extrêmes 27-95 points). Dix-sept patients (43 %) avaient une fatigabilité musculaire initiale sévère (SMM initial ≤ 50 points). La Figure 2 illustre la répartition des patients selon la sévérité de la fatigabilité musculaire initiale par le score SMM.

**Figure 3:**
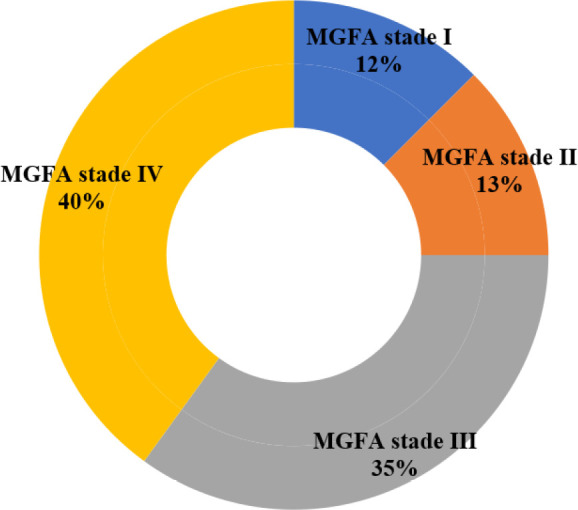
Répartition des patients selon l'importance de la fatigabilité musculaire initiale par le score SMM

Lors de l'examen clinique initial, l’évaluation du stade de gravité par le score MGFA a retrouvé 16 patients (40 %) au stade de myasthénie généralisée sévère (stade IV). La Figure 3 illustre la répartition des patients selon le stade de gravité clinique. Les dosages des Ac-anti-RACh et des Ac anti-MuSK ont été réalisés chez 33 patients sur 40 (83 %) et sont revenus positifs, respectivement dans 22 cas (67 %) et dans 4 cas (12 %). Ils étaient négatifs chez 7 patients (21 %). Ce dosage n'a pas pu être réalisé chez 7 patients. Le dosage des Ac-anti-MuSK est revenu positif chez les 4 patients sur les 11 cas séronégatifs pour les Ac anti-RACh. L’ENMG de stimulation nerveuse répétitive a été réalisée chez 39 patients et a mis en évidence un décrément d'amplitude du potentiel d'action musculaire significatif chez 34 d'entre eux (87 %). Une TDM thoracique réalisée chez 38 patients a retrouvé une hyperplasie du thymus chez 22 patients (58 %), un thymome chez 6 patients (16 %) et un thymus normal chez 10 patients (26 %). Un examen anatomopathologique des pièces d'exérèse de thymus après thymectomie chez 16 patients (40 %) a confirmé un thymome chez 6 d'entre eux. Le délai médian du diagnostic et de mise en route du traitement médicamenteux était de 22 mois (IQ=12, extrêmes 1 mois et 18 ans). Tous nos patients ont été traités par anticholinestérasiques oraux pendant une durée médiane de 36 mois (IQ=48, extrêmes 2 et 60 mois) : pyridostigmine bromure (Mestinon) chez 30 patients (75 %) et chlorure d'ambénonium (Mytelase^®^) chez 10 patients (25 %). En tout, 36 patients ont bénéficié d'un traitement de fond à visée immunologique par voie orale, soit par corticoïdes (prednisone ou prednisolone ou deflazacort) chez 25 patients, soit par immunosuppresseur (azathioprine) chez 5 patients, soit par une association corticoïde-azathioprine chez 10 patients. La durée médiane du traitement de fond était de 21 mois (IQ =22, extrêmes 1-252 mois). Un traitement adjuvant par supplémentation potassique et calcique et par inhibiteur de la pompe à protons, a été institué chez tous les patients mis sous corticothérapie. Une prise de poids excessive dans 12 cas, des infections itératives (cutanées, ORL) dans 8 cas, une gastrite érosive dans 6 cas, ont été les effets indésirables cortico-induits les plus fréquemment rencontrés (Tableau I).

**Tableau I:** Répartition des patients traités par corticoïdes selon les effets secondaires survenus sous traitement

Effets indésirables des corticoïdes	Effectifs (n=35)	Pourcentage (%)
Prise de poids excessive	12	30
Infections itératives	8	20
Gastrite érosive	6	15
Hyperglycémie	5	12
Hypertension artérielle	4	10
œdèmes des membres inférieurs	3	7
Dépigmentation cutanée	3	7
Alopécie	1	2

**Figure 4:**
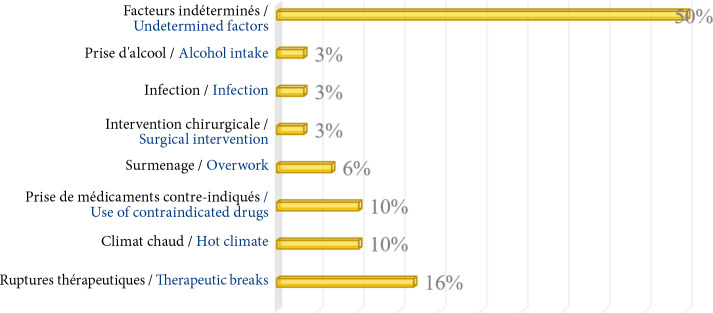
Répartition des patients selon les facteurs déclenchants des poussées de myasthénie

Au total, quatre patients sur neuf ont bénéficié pour crise myasthénique d'une cure d'immunoglobulines IV ou d’échanges plasmatiques. Une thymectomie a été pratiquée chez 16 des 27 patients qui avaient une hyperplasie thymique ou un thymome à la TDM. Les suites chirurgicales ont été satisfaisantes chez tous ces patients avec une durée médiane d'hospitalisation de 6 jours (IQ=2, extrêmes 4 et 11 jours). Le délai médian entre le diagnostic et la thymectomie a été de 18 mois (IQ=31, extrêmes 2 et 120 mois). L’évolution clinique a été marquée par la survenue d'une médiane de 3 poussées par an (IQ=2, extrêmes 1 et 30 poussées annuelles) chez 30 patients, dans un délai médian de 6 mois (IQ=5, extrêmes 1 et 48 mois), après le diagnostic. Les facteurs déclenchants, identifiés chez 15 patients, étaient liés en majorité aux ruptures thérapeutiques (Fig. 4).

**Figure 5:**
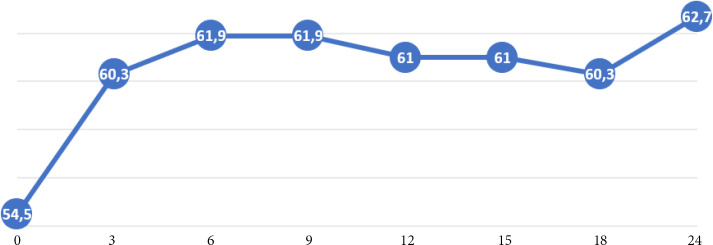
Courbe évolutive de la fatigabilité musculaire selon le SMM des patients atteints de MAI sous traitement médicamenteux exclusif sur 24 mois

Neuf patients ont présenté au moins une crise de myasthénie favorisée par une infection dans 6 cas ou par une rupture thérapeutique dans 3 cas. Sous traitement médicamenteux par anticholines-térasique, corticoïdes et/ou azathioprine, le profil évolutif de la fatigabilité musculaire a montré un gain du SMM médian de 6 points (IQ=2) par semestre (extrêmes 1 et 9 points) (Fig. 5).

**Figure 6:**
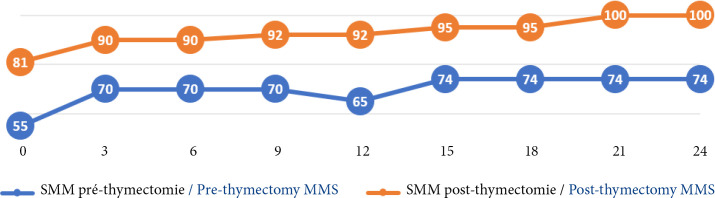
Comparaison des courbes évolutives du SMM moyen sur 24 mois, en pré et post thymectomie chez les patients atteints de MAI ayant bénéficié d'une thymectomie chirurgicale

Chez les 16 patients ayant bénéficié d'une thymectomie, la comparaison des courbes de la fatigabilité musculaire selon le SMM a montré que le nombre médian de points gagnés par semestre était de 5 points (IQ=39, extrêmes 27-35 points) en préthymectomie et de 5 points également (IQ=19, extrêmes 11-20 points) en post thymectomie. Cependant le traitement médicamenteux aboutissait à une régression partielle de la fatigabilité musculaire, alors que la thymectomie s'accompagnait d'une régression complète de la fatigabilité musculaire après un délai de 24 mois (Fig. 6).

**Figure 7:**
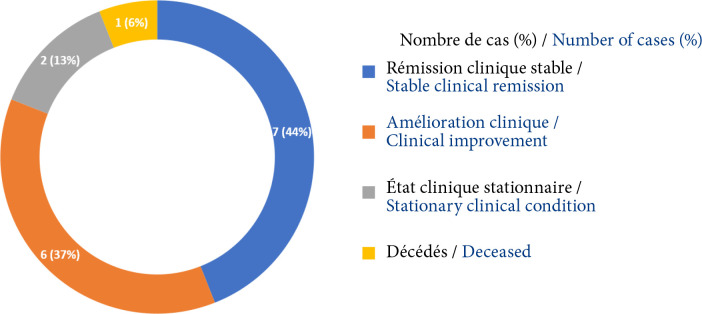
Répartition des patients selon l'évolution clinique en fin de suivi après thymectomie

**Figure 8:**
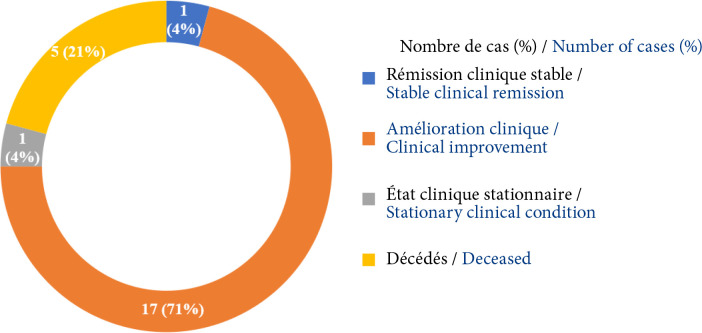
Répartition des patients n'ayant bénéficié que du traitement médicamenteux, selon l'évolution clinique en fin de suivi

À la fin de l’étude, après un suivi ambulatoire médian de 53 mois (IQ=16, extrêmes 12 mois et 120 mois) :

- parmi les 16 patients ayant bénéficié d'une thymectomie, 7 étaient en rémission clinique stable, 6 étaient cliniquement améliorés, 2 étaient restés stationnaires et 1 était décédé (Fig. 7).
- parmi les 24 patients n'ayant bénéficié que du traitement médicamenteux, un seul était en rémission clinique stable, 17 étaient cliniquement améliorés, 1 était resté stationnaire et 5 étaient décédés (Fig. 8).

**Figure 9:**
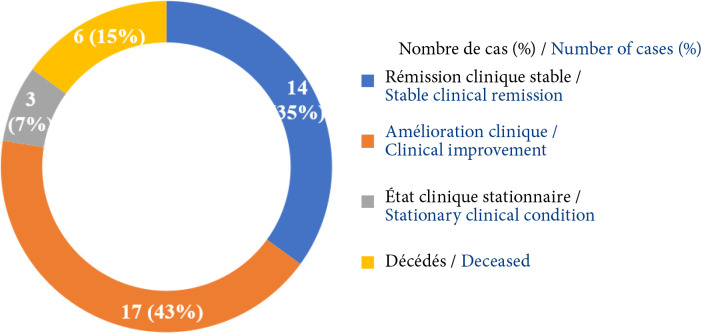
Répartition des patients atteints de MAI selon l'évolution clinique en fin de suivi

À la fin de l’étude, sur l'ensemble des 40 patients : 6 étaient décédés (soit un taux de mortalité de 15 %), 14 étaient en rémission clinique stable (35 %), 17 étaient cliniquement améliorés (43%) et 3 (7 %) avaient un état clinique stationnaire (Fig. 9).

La cause immédiate des décès a été dans tous les cas une crise myasthénique, survenue dans un contexte de rupture thérapeutique (par inaccessibilité financière ou non disponibilité des médicaments) et de surinfection bronchopulmonaire favorisée par une immunodépression iatrogénique (emploi au long cours des corticoïdes ou immunosuppresseurs), ayant nécessité une hospitalisation en réanimation.

## Étude analytique

Après analyse bivariée, un stade de gravité clinique initial sévère de la MAI a été identifié comme le seul facteur associé à une évolution clinique défavorable (MGFA de stade IV ou V ou décès, p=0,025). Cf. Tableau II.

**Tableau II:** Analyse bivariée entre les variables générales et l'évolution clinique en fin d'étude des patients suivis pour MAI

Variables	Modalités	Défavorable	Favorable	p
Âge	≤50 ans	6	25	0,12
	>50 ans	4	5	
Sexe	Féminin	7	17	0,45
	Masculin	3	3	
Âge médian de début (ans)		28	25	0,71
Délai médian de diagnostic (mois)		13	10	0,33
Trouble de la deglutition	Oui	7	17	0,45
	Non	3	3	
SMM médian initial		36	16	0,06
Délai médian entre le diagnostic et le début du traitement		1	1	0,47
Délai médian entre les 1ers signes et le diagnostic		13	10	0,33
Délai médian entre le début de la maladie et le traitement chirurgical		14	10	0,11
Délai médian entre le diagnostic et la thymectomie		11	24	0,52
Classe MGFA initiale	MGFA≥4	7	9	0,02
	MGFA>4	3	21	
Ac anti-RACh	Oui	5	17	0,71
	Non	5	13	
Anomalie du thymus (hyperplasie ou thymome)	Oui	7	21	1,00
	Non	3	9	
Thymectomie Thymectomy	Oui	2	14	0,13
	Non	8	16	

## Discussion

La prise en charge des patients myasthéniques est habituellement confrontée à un retard diagnostique, un peu partout dans le monde, mais surtout en Afrique subsaharienne. Le délai diagnostique était de 26 mois à Ouagadougou au Burkina Faso en 2019 [23], 24 mois au Sénégal [19, 47]. Ce retard concerne 80 % des patients en Italie [27], et excède 24 mois chez plus 50 % des patients aux Pays-Bas [4]. *A contrario*, des délais diagnostiques relativement plus courts, (2 mois en Corée du Sud [31] et 11 mois au Kenya [7]), ont été rapportés. Ces retards peuvent découler dans notre contexte de la non-perception par les patients du potentiel de gravité évolutive de la MAI au stade débutant oculaire. La longueur de l'itinéraire diagnostique et thérapeutique, favorisée par la complexité diagnostique, par l'insuffisance du nombre de neurologues et par les difficultés d'accès aux explorations et aux traitements adéquats peuvent aussi y participer [7, 23]. Afin de contribuer au diagnostic précoce de la MAI notamment dès le stade oculaire, nous préconisons à la suite de Bundi Karau *et al.* [7], une collaboration accrue entre ophtalmologistes et neurologues.

Les anticholinestérasiques (pyridostigmine bromure, chlorure d'ambénonium), traitement symptomatique de base de la MAI [16, 17, 24], ont été utilisés chez tous les patients de notre série. Leur usage est parfois limité par leurs effets indésirables muscariniques ou nicotiniques, toutefois transitoires [17, 25, 28, 32, 33, 37], retrouvés chez 35 % de nos patients.

Dans notre série, 92 % des patients dont les symptômes n’étaient pas suffisamment ou durablement améliorés par les anticholinestérasiques, ont bénéficié d'un traitement de fond à visée immunologique soit par corticoïdes (62 %), soit par immunosuppresseurs de type azathioprine (12 %), rarement avec association des 2 thérapeutiques (17 %). Cette stratégie thérapeutique est conforme aux recommandations en vigueur [17, 24, 39]. Les autres immunosuppresseurs tels le mycophénolate mofetyl, la ciclosporine, le tacrolimus, le cyclophosphamide, et plus récemment le rituximab, non utilisés car non disponibles et non accessibles financièrement dans notre contexte, constituent des alternatives thérapeutiques [8, 12, 17].

Les EP et les IgIV polyvalentes constituent les seuls traitements spécifiques d'efficacité prouvée, indiqués lors des poussées myasthéniques aiguës sévères incluant les crises myasthéniques, ou en préparation d'une thymectomie lorsque la myasthénie reste franchement symptomatique et/ ou mal équilibrée. Le choix entre ces deux types de traitement dépend de leurs contre-indications respectives et de l'offre de l'hôpital. Les IglV d'utilisation souvent plus pratique et avec un risque moindre d'effets secondaires sévères, sont souvent préférées aux EP malgré les délais d'efficacité légèrement plus courts des EP [17, 32, 34, 43]. La très faible accessibilité financière des IglV pour nos patients et l'indisponibilité des EP dans la plupart des hôpitaux d’Afrique subsaharienne, notamment au Bénin, Gabon, Sénégal, Nigeria et Burkina Faso [23], limitent leur utilisation lors des poussées sévères ou des crises myasthéniques. Ainsi dans notre série, les taux d'utilisation des IglV et des EP ont été respectivement de 8 % et 0 % malgré la survenue de crises myasthéniques chez 22 % de nos patients. De même en Côte-d’Ivoire, aucun des 5 patients n'a pu en disposer faute de moyens financiers [11]. Par contre à Trinidad et Tobago [2, 26], respectivement, 11 % et 40 % des patients ont bénéficié des EP et des IgIV.

De nombreuses études ont rapporté un bénéfice substantiel de la thymectomie dans les MAI [14, 41]. Son efficacité *versus* un traitement médicamenteux dans la MAI avec ou sans anomalie thymique a été démontrée récemment à travers plusieurs études. Il s'agit de l'essai randomisé MGTX réalisé dans 36 centres à travers le monde entre 2006 et 2012 [45], d'une étude rétrospective à partir d'une base de données prospective de 3 017 patients entre 1941 et 2013 à New York [20], et d'une revue systématique de la littérature publiée sur MEDLINE jusqu'en juin 2015 [42]. Ces études ont permis de conclure à la supériorité de la thymectomie par rapport au traitement médicamenteux conservateur sur la rémission de la MAI avec ou sans hyperplasie thymique et d’étendre l'indication de la thymectomie à tous les patients atteints de MAI lorsque cela est possible [20]. Cependant les critères de sélection des patients, l'efficacité de la thymectomie sur les différents sous-types de MAI [32], les délais de l'intervention chirurgicale et la technique de résection optimale, restent encore à préciser [40]. Dans notre série, une rémission clinique stable avec arrêt des médicaments, ou une rémission pharmacologique avec poursuite des médicaments, ont été observées chez 44 % des patients ayant bénéficié d'une thymectomie pour thymome ou hyperplasie thymique *versus* 4 % des patients qui n'ont bénéficié que du traitement médicamenteux, après un suivi médian post-thymectomie de 53 mois. Nos résultats confirment ainsi la supériorité d'efficacité de la thymectomie *versus* un traitement médicamenteux, malgré les retards dans sa mise en œuvre.

En Afrique subsaharienne, les séries publiées rapportent une évolution clinique au long cours peu favorable marquée par un faible taux de rémission complète stable, de 20 % au Sénégal dans une série pédiatrique [38], 6,3 % au Kenya [7], 5,5 % à Trinidad et Tobago [26], et aucun patient à Madagascar [36], *versus* 35 % de l'ensemble de nos patients, proche des 40 % observés en Lituanie [35]. La modalité évolutive clinique la plus fréquemment rapportée est une régression partielle de la symptomatologie (amélioration clinique), constatée chez 68,8 % des patients au Kenya [7], 57,1 % à Trinidad et Tobago [26] et à Madagascar [36], 60 % au Sénégal [38], *versus* 43 % de l'ensemble des patients de notre série. Une résistance aux thérapeutiques marquée par l'absence de toute amélioration clinique (ou état clinique stationnaire) malgré le traitement, est également assez fréquemment observée en ASS : 28 % des patients à Madagascar [36], 10 % au Sénégal [38], 7 % au Kenya [7] et 7 % chez les patients de notre série. Plusieurs facteurs expliquent cette évolution clinique au long cours globalement défavorable de la MAI dans le contexte de l’ASS : les retards diagnostiques aux stades sévères de la maladie dus à la longue errance des patients, les difficultés diagnostiques et de prise en charge de la maladie liées à la non-disponibilité et/ou à l'inaccessibilité financière des explorations paracliniques (dosages des auto Ac) et des thérapeutiques d'efficacité prouvée pour les patients. En ASS, les patients atteints de MAI n'ont pas accès, dans leur grande majorité, à la thymectomie, faute de plateaux techniques adéquats ou de disponibilité de chirurgiens thoraciques [7, 36, 38]. Dans notre série, 40 % des patients ont tout de même bénéficié d'une thymectomie en partie grâce à la disponibilité d'un plateau technique et de trois chirurgiens thoraciques.

Dans les pays développés, grâce à la disponibilité de moyens diagnostiques performants, de thérapeutiques à visée immunologique d'urgence d'efficacité prouvée comme les IglV ou les EP, de thérapeutiques immunomodulatrices au long cours comme la thymectomie et les anticorps monoclonaux, associées à des mesures de réanimation adéquates, la mortalité de la MAI a considérablement diminué à moins de 5 % de nos jours. Les décès actuels sont liés à la sévérité de l'atteinte respiratoire et aux complications des traitements [13, 32, 35]. En Chine, des taux de mortalité de 1,5 % ont même récemment été rapportés [9]. En ASS, les taux de mortalité restent encore assez élevés, variant de 27,5 % au Nigéria [30], 16,7 % en intra-hospitalier et 25 % lors du suivi au long cours au Kenya [7], 15 % dans notre série, 14 % à Madagascar [36], 10 % chez les enfants au Sénégal [38]. Ces forts taux de mortalité dans notre contexte s'expliquent par la non-disponibilité et/ou la non-accessibilité financière des traitements pour la prise en charge des crises ou des poussées myasthéniques sévères, rapidement mortelles. La non-disponibilité des anticorps monoclonaux comme le rituximab pour la prise en charge des cas réfractaires, les retards diagnostiques au stade généralisé sévère de la maladie, constituent également des facteurs de risque de décès des patients myasthéniques en ASS [22, 23, 44].

Dans notre série, seul le diagnostic de la MAI au stade généralisé sévère était significativement associé (p=0,02) à une évolution défavorable définie par la constatation d'un stade clinique sévère à très sévère (stade IV ou V de la MGFA) ou la survenue d'un décès lié à la MAI en fin de suivi. Lorsque l'on considère la mortalité associée à la MAI, celle-ci est étroitement liée à l'atteinte respiratoire et à l'atteinte des muscles d'innervation bulbaire, spécifiques du stade généralisé sévère [5, 21, 46]. En revanche, plusieurs études ont identifié également la MAI à début tardif et la coexistence de la MAI avec d'autres maladies auto-immunes, comme principaux facteurs de risque d’évolution défavorable [3, 5, 10, 13, 21, 29, 46]. La MAI à début tardif s'accompagne d'un taux plus élevé de thymomes [5, 29]. Les patients plus âgés ont moins de chances d'obtenir une rémission complète et stable [3] et sont plus susceptibles de souffrir d'une crise myasthénique avec un pronostic défavorable, y compris le décès [10]. Leur prise en charge est également difficile du fait des comorbidités et d'un risque plus élevé d'effets secondaires liés à l'acétylcholinestérase et aux corticoïdes [13]. Par ailleurs, plusieurs études ont identifié 3 facteurs de risque de généralisation secondaire de la MAI : l'hyperplasie thymique, la séropositivité aux Ac spécifiques de la MAI et les comorbidités auto-immunes [46]. D'autres études ont démontré qu'une exposition précoce et prolongée des patients aux thérapeutiques immunosuppressives (particulièrement la corticothérapie), était le principal facteur protecteur contre l'aggravation de la maladie [5, 21, 46]. Ainsi un diagnostic précoce, un meilleur accès et une disponibilité accrue des moyens diagnostiques et thérapeutiques, l'immunothérapie au long cours (notamment la thymectomie, les immunosuppresseurs, les anticorps monoclonaux) permettraient de réduire la mortalité. Une subvention étatique, une éducation des patients qui s'appuierait sur des associations de patients, aideraient à améliorer le pronostic fonctionnel et la qualité de vie des patients suivis pour MAI en ASS.

## Limites et avantages de notre étude

Le faible effectif de nos patients, l'absence de dosages des Ac anti-RACh, des Ac anti-MuSK chez une proportion relativement importante de nos patients, faute de moyens financiers, a pu induire des biais.

De même, le caractère hospitalier de notre étude a pu constituer un biais de sélection des cas les plus graves aux dépens des formes cliniques oculaires pures ou généralisées légères.

Ces limites sont largement contrebalancées par les avantages de cette étude : suivi longitudinal médian de 53 mois (IQ=10, extrêmes 12 et 120 mois); diagnostic de MAI basé sur un faisceau d'arguments cliniques, paracliniques et/ou thérapeutiques; dosages des Ac anti-RACh ou anti-MuSK réalisés dans le même laboratoire d'analyses bio médicales; examens d’ENMG effectués sur le même type d'appareils; proportion importante de patients ayant bénéficié d'une thymectomie dans le même centre par la même équipe chirurgicale.

## Conclusion

Au Burkina Faso, la MAI est tributaire d'un retard diagnostique. La quasi-totalité des patients suivis pour MAI bénéficie d'un traitement au long cours par anticholinestérasiques et corticoïdes seuls, ou associés à l'azathioprine. Bien que disponible, la thymectomie y est d'accessibilité financière limitée avec de longs délais d'attente. L’évolution clinique est fréquemment émaillée de poussées et crises myasthéniques. La létalité reste élevée puisqu'elle concerne environ 1 cas sur 10. La rémission clinique ne concerne qu'environ 1/3 des patients survivants, du fait d'une non-disponibilité et d'une inaccessibilité financière des traitements. Un meilleur accès et une disponibilité accrue des médicaments anti myasthéniques, grâce notamment à une subvention étatique, une éducation thérapeutique des patients, une organisation des patients en associations, permettront de réduire la morbi-mortalité et d'améliorer le pronostic fonctionnel des patients suivis pour MAI dans notre contexte.

## Sources de financement

L'étude n'a bénéficié d'aucun financement.

## Contribution des auteurs

LOMPO Djingri Labodi : conception de l’étude, recherche documentaire, recueil des données, élaboration du protocole de l’étude, analyse des données, rédaction du manuscrit.

NACOULMA Hervé : recueil des données, analyse des données, recherche documentaire. KYELEM Julie Marie Adeline : recueil des données, révision et validation du protocole, révision du manuscrit.

ZOUNGRANA Alassane : recueil des données, validation du protocole.

KERE M Fabienne Yabtouta : recueil des données, rédaction du manuscrit.

GNAMPA Melody Zeinab : recueil des données. NAPON Christian : validation du protocole de l’étude, supervision de l’étude.

MILLOGO Athanase : validation du protocole de l’étude, supervision de l’étude.

## Conflit d'intérêt

Les auteurs ne déclarent aucun conflit d'intérêt.
