# Supplementary material for: Treatment and clinical course of autoimmune myasthenia in Burkina Faso
Source: Med Trop Sante Int. 2025 Feb 21;5(1):mtsi.v5i1.2025.646. [Article in French] doi: 10.48327/mtsi.v5i1.2025.646 (PMC12001994; doi:10.48327/mtsi.v5i1.2025.646)
Supplement: Related texts

# Research article: Treatment and clinical course of autoimmune myasthenia in Burkina Faso

## Introduction

Myasthenia *(myasthenia gravis*, AMG) is an autoimmune disease of the neuromuscular junction, characterized by muscular fatigue and weakness that appear or worsen with exertion and diminish or disappear with rest. It is due to the presence of specific autoantibodies directed against postsynaptic membrane proteins of the neuromuscular junction, mainly anti-nicotinic acetylcholine receptor antibodies (anti-RACh antibodies) and anti-muscle specific kinase antibodies (anti-MuSK antibodies) [6, 14, 17]. The clinical and paraclinical manifestations are highly heterogeneous and vary according to the muscle territories affected, the age of onset of symptoms, the type of autoantibodies or any associated thymic abnormalities. Initial manifestations are mainly ocular, with ptosis and diplopia occurring in over 67% of cases [15.17.39.48]. The disease progresses to affect other areas, particularly the respiratory, swallowing, and limb muscles in more than 80% of cases, with a rapid increase in severity indicating a myasthenic crisis [1, 3]. The severity of myasthenia lies in the involvement of the respiratory muscles. Early, multidisciplinary care with current therapies - particularly corticosteroid therapy, immunotherapy and thymectomy on the one hand, and plasma exchange (PE), immunoglobulin IV (IVIG) and rituximab on the other - has significantly reduced mortality and improved patients’ functional prognosis and quality of life [15.17.39.48] . In Sub-Saharan Africa (SSA), AMG is poorly understood, underdiagnosed, characterized by delayed diagnosis and poor availability and accessibility of proven effective diagnostic and therapeutic tools, resulting in poor functional prognosis and still high mortality [7, 23]. The aim of the present study is to describe the therapeutic modalities and evaluate the clinical evolution of AMG in Burkina Faso through a longitudinal descriptive and analytical study.

## Patients and methods

This was a longitudinal, descriptive, multicenter, hospital-based study involving all private and public health facilities in the 13 health regions of Burkina Faso. The study covered a period of 5 years and 6 months (March 2015 to September 2019). Before the start of the study, official correspondence was sent to all physicians registered with the Burkina Faso Medical Association and working in the various public health facilities (46 medical centers, 13 regional hospital centers, 5 university hospital centers) and private facilities (350 clinics and private practices). Correspondence was also sent to the administrative heads of the aforementioned health facilities (13 regional health directors, 70 chief medical officers of health districts), asking them to report any suspected cases of myasthenia (patients with clinical symptoms suggestive of myasthenia) or confirmed cases of myasthenia. Out of a total of 368 health facilities in Burkina Faso, we contacted 218 (7 hospitals, 11 urban medical centers, and 200 private health facilities), of which 199 cooperated and 36 referred suspected cases to us. Confirmed cases came from four hospitals, two urban health centers and three private clinics. Patients referred to us were regularly seen as neurology outpatients at the three main university hospitals in Ouagadougou: CHU Tingandogo (CHU-T), CHU Yalgado Ouédraogo, and CHU de Bogodogo. Patients who were unable to travel to the study site were seen in their own facilities after express travel by the investigating neurologist team.

All patients with clinical symptoms suggestive of AMG associated with at least one of the following criteria were included in our study:

- Presence in serum of anti-RACh antibodies at a level > 0.4 nmol/mL (negative or doubtful if ≤ 0.4 nmol/mL) and/or anti-MuSK antibodies;
- Presence of a significant decrease in muscle action potential amplitude (> 10%) between the 1^st^ and 5^th^ stimulation in at least 2 nerve-muscle pairs during repetitive stimulation at 3 cycles/second by nerve stimulation electroneuromyography, after discontinuation of anticholinesterase drugs the day before the examination;
- Therapeutic trial with oral anticholinesterase drugs [pyridostigmine bromide, 60 mg tablets (Mestinon®) or ambenonium chloride, 10 mg tablets (Mytelase®)] showing a clear functional benefit in the patient's daily life for at least 14 days.

A total of 78 cases of suspected myasthenia benefited from therapeutic trials, mainly with pyridostigmine bromide tablets 60 mg *per os* every 4 hours from waking to bedtime for at least 14 days. A total of 96 patients were referred for suspected myasthenia.

Patients whose clinical symptoms were suggestive of myasthenia but not confirmed by paraclinical tests and/or patients who did not respond to therapeutic testing with oral anticholinesterase agents were not included in the study.

Since anti-RACh and anti-MuSK antibodies assays are not available in Burkina Faso, all serum samples for these assays were collected and packaged by a team of experienced biologists at a single reference laboratory in Ouagadougou (Laboratoire Bio 2000), which has a collaborative agreement with a reference laboratory in France. Samples were sent to the CERBA medical biology laboratory for analysis by radioimmunoassay for anti-RACh antibodies and by enzyme-linked immunosorbent assay (ELISA) for anti-MuSK antibodies.

Electromyoneurography (EMNG) was performed in the clinical neurophysiology laboratories of Tengandogo and Bogodogo Teaching Hospitals using the same type of Neurosoft® equipment. For each examination, the following nerve-muscle pairs were studied bilaterally: ulnar-abductor *digiti minimi*, median-abductor *pollicis brevis*, axillary-deltoid, *facial-orbicularis oculi.*

On admission, a medical history was taken of each patient, including past medical history, diagnostic history, associated antecedents and comorbidities, habits, and lifestyle. A clinical examination was then performed, including assessment of general condition, vital signs, muscle fatigability using the initial Motor Muscle Score (MMS), and an initial assessment of clinical severity using the Myasthenia Gravis Foundation of America (MGFA) staging system. Similarly, each patient underwent a paraclinical workup on admission, including repetitive nerve stimulation EMNG, anti-RACh and anti-MuSK antibodies serum assay, thyroid hormones assay, serum autoimmunity check, and chest CT scan. However, due to the cost, only some patients were able to undergo these various tests due to lack of financial resources. Patients were then started on symptomatic anticholinesterase therapy. Dependig on clinical response, patients were then treated with oral corticosteroids or azathioprine. If the response was inadequate, corticosteroids and azathioprine were combined. Thymectomy with anatomopathologic examination of the excision specimen has been proposed for certain patient profiles (young AMG, anti-RACh antibody seropositivity, with thymic hyperplasia or AMG associated with a thymoma). Some patients have benefited from this approach because they have sufficient financial resources. Other treatments for AMG were not yet available in our setting.

After enrollment, each patient benefited from long-term longitudinal follow-up with neurological clinical evaluations every 3 months for 12 months, then every 6 months. The occurrence of myasthenic relapses or attacks and the context in which they occurred, the occurrence of treatment-related adverse events, and any other events were regularly reported and managed. MMS and clinical progression according to MGFA stages were also regularly recorded.

At the end of the study, patients were divided into survivors and decedents, and survivors were classified according to functional prognosis.

The study variables included were:

- Sociodemographic: age, sex, time to neurology consultation;
- Clinical: type of onset (acute/subacute/progressive), time to diagnosis, initial MMS, MGFA stages;
- Paraclinical: determination of anti-RACh and anti-MuSK antibodies, chest CT, EMNG, anatomopathologic examination of excised specimens;
- Therapeutic: time to start treatment, molecules used, side effects, thymectomies performed, etc.
- Evolution: number of relapses, number of myasthenic attacks, favoring context; evolution of MMS progression indices; evolution of clinical severity according to MGFA stages; mortality; clinical outcome of survivors.

These data were analyzed using Epi-Info 7.2.5.0 software. Data were presented as percentages for qualitative variables and as means and percentages for quantitative variables. Student's t-tests and chi-squared tests were used when appropriate. Bivariate analysis with calculation of p-value (<0.05) was used to identify factors associated with unfavorable clinical outcome.

An unfavorable clinical course was defined as a stationary or worsening clinical condition or death at the end of follow-up.

## Operational definitions

A myasthenic crisis was defined as a myasthenic attack involving the respiratory or bulbar muscles responsible for respiratory distress requiring mechanical ventilation and immediate care in an intensive care unit.

A cholinergic crisis was defined as the combination of muscarinic signs (diarrhea, hypersalivation, bronchial hypersecretion, sweating) and nicotinic signs (fasciculations, convulsions, Chvostek's sign) in a patient overtreated with anticholinesterase agents.

The MMS, scored from 0 to 100 points, was used to assess muscular fatigability by measuring muscle strength in different parts of the body during the initial clinical examination and subsequently during follow-up visits.

At the initial clinical examination, patients were divided into five subgroups of increasing clinical severity according to the MGFA classification [16, 19]. The “MGFA post-intervention status” classification, adapted to our context, was used to assess patient outcome at the end of follow-up. This assessment identified five possible evolutionary modalities at the end of the study, each exclusive of the others:

- Complete and stable remission or pharmacological remission: complete disappearance of signs and symptoms of AMG for at least one year, with or without continuation of antimyasthenic drugs;
- Clinical improvement: significant reduction of the manifestations observed before treatments. Only change from at least one MGFA class to a lower class was considered;
- Stable or unchanged clinical status: no significant change in manifestations observed before treatments;
- Clinical worsening: significant increase in manifestations, occurrence of myasthenic relapses or attacks despite treatment, or change from at least one less severe MGFA class to a more severe one;
- Death.

The study was approved by the National Ethics Committee of Burkina Faso. Data collection was carried out with the approval of the administrations of the various health facilities. Data collection forms were completed on site after obtaining the consent of the patients or their legal guardians. The data collected were kept confidential.

## Results

During the study period, 40 patients were enrolled, including 24 females (60%) and 16 males (40%), with a sex ratio of 1.5. The median age of onset was 25 years (IQ=7, extremes 4 and 71 years). There were 5 cases of childhood-onset AMG (13%), 32 cases of young adult-onset AMG (80%), and 3 cases of elderly-onset AMG (7%). The median delay in consulting a neurologist was 21 months (IQ=12, extremes 1 month and 18 years). The median time to diagnosis was 22 months (IQ=12, extremes 1 month and 217 months). The clinical presentation was dominated by oculomotor or visual disturbances, phonation disorders, limb muscle weakness, and dyspnea due to respiratory muscle damage in 36 patients (90%), 28 patients (70%), 27 patients (68%), and 26 patients (65%), respectively (Fig. 1).At the initial clinical examination, muscle fatigability was assessed by a median MMS of 57 points/100 (IQ=35, extremes 27-95 points). Seventeen patients (43%) had severe initial muscle fatigability (initial MMS ≤ 50 points). Figure 2 illustrates the distribution of patients according to the severity of initial muscle fatigability by MMS score.

**Figure 1:** Distribution of patients according to the clinical presentations revealing autoimmune myasthenia in the 40 patients

**Figure 2:** Distribution of patients according to the extent of initial muscle fatigability by MMS score

At the initial clinical examination, 16 patients (40%) were in the stage of severe generalized myasthenia (stage IV) according to the MGFA score. Figure 3 shows the distribution of patients according to clinical severity.

**Figure 3:** Distribution of AMG patients according to initial clinical severity (MGFA classification) at diagnosis

Anti-RACh and anti-MuSK antibodies assays were performed in 33 of 40 patients (83%) and were positive in 22 (67%) and 4 (12%) cases, respectively. They were negative in 7 patients (21%). The assay could not be performed in 7 patients. The anti-MuSK antibody assay was positive in 4 of the 11 anti-RACh antibody negative cases. EMNG repetitive nerve stimulation was performed in 39 patients and showed a significant decrease in muscle action potential amplitude in 34 patients (87%).

Chest CT scans in 38 patients showed thymic hyperplasia in 22 (58%), thymoma in 6 (16%), and normal thymus in 10 (26%). Pathologic examination of the thymus removed after thymectomy in 16 patients (40%) confirmed thymoma in 6 patients.

The median time to diagnosis and initiation of medical therapy was 22 months (IQ=12, extremes 1 month and 18 years). All patients were treated with oral anticholinesterase agents for a median of 36 months (IQ=48, extremes 2 and 60 months): pyridostigmine bromide (Mestinon®) in 30 patients (75%) and ambenonium chloride (Mytelase®) in 10 patients (25%). A total of 36 patients received oral immunosuppressive therapy, either corticosteroids (prednisone or prednisolone or deflazacort) in 25 patients, immunosuppressants (azathioprine) in 5 patients, or a combination of corticosteroids and azathioprine in 10 patients. The median duration of background treatment was 21 months (IQ=22, range 1-252 months). Adjuvant treatment with potassium and calcium supplements and proton pump inhibitors was initiated in all patients receiving corticosteroid therapy. Excessive weight gain in 12 cases, recurrent infections (skin, ENT) in 8 cases, and erosive gastritis in 6 cases were the most common cortico-induced adverse events (Table I).

**Table I:** Distribution of patients treated with corticosteroids according to treatment-emergent side-effects

Side effects of corticosteroids	Headcount (n=35)	Percentage (%)
Excessive weight gain	12	30
Recurrent infections	8	20
Erosive gastritis	6	15
Hyperglycemia	5	12
High blood pressure	4	10
Edema of the lower limbs	3	7
Skin depigmentation	3	7
Alopecia	1	2

Four of nine patients underwent intravenous immunoglobulin therapy or plasma exchange for myasthenic crisis. Thymectomy was performed in 16 of the 27 patients with thymic hyperplasia or thymoma on CT. Surgical follow-up was satisfactory in all patients, with a median hospital stay of 6 days (IQ=2, extremes 4 and 11 days). The median time from diagnosis to thymectomy was 18 months (IQ=31, extremes 2 and 120 months). The clinical course was characterized by a median of 3 relapses per year (IQ=2, extremes 1 and 30 annual relapses) in 30 patients, with a median delay of 6 months (IQ=5, extremes 1 and 48 months) after diagnosis. The precipitating factors identified in 15 patients were mostly related to therapeutic breakthroughs (Fig. 4).

**Figure 4:** Distribution of patients according to triggers for myasthenia gravis attacks

Nine patients had at least one crisis of myasthenia precipitated in 6 cases by an infection and in 3 cases by a therapeutic break.

Under treatment with anticholinesterase, corticosteroids and/or azathioprine, the evolution profile of muscle fatigability showed a median MMS gain of 6 points (IQ=2) per half-year (extremes 1 and 9 points) (Fig. 5).

**Figure 5:** Evolutionary curve of muscular fatigability according to MMS in AMG patients on exclusive drug treatment over 24 months

In the 16 patients who underwent thymectomy, comparison of the muscle fatigability curves according to MMS showed that the median number of points gained per half-year was 5 points (IQ=39, extremes 27-35 points) before thymectomy and also 5 points (IQ=19, extremes 11-20 points) after thymectomy. However, drug treatment resulted in a partial regression of muscle fatigability, whereas thymectomy was associated with a complete regression of muscle fatigability at 24 months (Fig. 6).

**Figure 6:** Comparison of 24-month mean MMS evolution curves, pre- and post-thymectomy in patients with AMG who underwent surgical thymectomy.

At the end of the study, after a median outpatient follow-up of 53 months (IQ=16, extremes 12 months and 120 months):

- Of the 16 patients who underwent thymectomy, 7 were in stable clinical remission, 6 were clinically improved, 2 remained stationary, and 1 had died (Fig. 7).
- Of the 24 patients who received medical therapy alone, only one was in stable clinical remission, 17 were clinically improved, 1 remained hospitalized, and 5 died (Fig. 8).

**Figure 7:** Evolutionary curve of muscular fatigability according to MMS in AMG patients on exclusive drug treatment over 24 months

**Figure 8:** Distribution of patients treated only by drugs according to clinical course at the end of follow-up

At the end of the study, 6 of the 40 patients had died, (mortality rate of 15%), 14 were in stable clinical remission (35%), 17 were clinically improved (43%), and 3 (7%) were clinically stable (Fig. 9). The immediate cause of death in all cases was a myasthenic crisis, which occurred in a context of therapeutic failure (due to financial inaccessibility or unavailability of drugs) and bronchopulmonary superinfection, favored by iatrogenic immunodepression (long-term use of corticosteroids or immunosuppressants), requiring hospitalization in an intensive care unit.

**Figure 9:** Distribution of AMG patients by clinical course at the end of follow-up

## Analytical study

After bivariate analysis, an initial severe clinical stage of AMG was identified as the only factor associated with an unfavorable clinical outcome (stage IV or V MGFA or death, p=0.025). See Table II.

**Table II:** Bivariate analysis between general variables and clinical course at the end of the study for patients followed for AMG

Variables	Conditions	Unfavorable	Favorable	p
Age	≤50 years	6	25	0,12
	>50 years	4	5	
Gender	Female	7	17	0,45
	Male	3	3	
Median age of onset (years)		28	25	0,71
Median time to diagnosis (months)		13	10	0,33
Swallowing disorder	Yes	7	17	0,45
	No	3	3	
Median initial MMS		36	16	0,06
Median time from diagnosis to start of treatment		1	1	0,47
Median time from 1st signs to diagnosis		13	10	0,33
Median time from onset to surgical treatment		14	10	0,11
Median time from diagnosis to thymectomy		11	24	0,52
Initial MGFA class	MGFA≥4	7	9	0,02
	MGFA>4	3	21	
Anti-RACh antibodies	Yes	5	17	0,71
	No	5	13	
Thymus abnormality (hyperplasia or thymoma)	Yes	7	21	1,00
	No	3	9	
Thymectomie Thymectomy	Yes	2	14	0,13
	No	8	16	

## Discussion

The management of myasthenic patients is usually confronted with a delay in diagnosis, almost everywhere in the world, but especially in sub-Saharan Africa. The diagnostic delay was 26 months in Ouagadougou, Burkina Faso, in 2019 [23], 24 months in Senegal [19, 47]. This delay affects 80% of patients in Italy [27] and exceeds 24 months in more than 50% of patients in the Netherlands [4]. In contrast, relatively shorter diagnostic delays have been reported (2 months in South Korea [31] and 11 months in Kenya [7]). In our context, these delays may be due to patients’ lack of awareness of the potential severity of AMG in the early ocular stage. The length of the diagnostic and therapeutic pathway, favored by the complexity of the diagnosis, the shortage of neurologists and the difficulty of access to appropriate investigations and treatment, may also play a role [7, 23]. Following Bundi Karau *et al.* [7], we recommend increased collaboration between ophthalmologists and neurologists to facilitate early diagnosis of AMG, especially during the ocular symptoms. Anticholinesterase agents (pyridostigmine bromide, ambenonium chloride), the basic symptomatic treatment for AMG [16, 17, 24], were used in all patients in our series. Their use is sometimes limited by their muscarinic or nicotinic side effects, which are nevertheless transient [17, 25, 28, 32, 33, 37] and were found in 35% of our patients.

In our series, 92% of patients whose symptoms were not sufficiently or durably improved by anticholinesterase agents received immunologic background treatment with either corticosteroids (62%) or azathioprine-type immunosuppressants (12%), rarely a combination of the 2 therapies (17%). This therapeutic strategy is in line with current recommendations [17, 24, 39]. Other immunosuppressive agents such as mycophenolate mofetil, cyclosporine, tacrolimus, cyclophosphamide and, more recently, rituximab, which are not used due to their unavailability and financial unaffordability in our context, are therapeutic alternatives [8, 12, 17].

PE and polyvalent IVIG are the only specific treatments with proven efficacy, indicated for severe acute myasthenic relapses, including myasthenic crisis, or in preparation for thymectomy when myasthenia remains openly symptomatic and/ or poorly balanced. The choice between these two types of treatment depends on their respective contraindications and hospital availability. IVIG, which is often more convenient to use and carries a lower risk of serious side effects, is often preferred to PE, despite the slightly shorter duration of action of PE [17, 32, 34, 43]. The very low affordability of IVIG for our patients and the unavailability of PE in most hospitals in sub-Saharan Africa, especially in Benin, Gabon, Senegal, Nigeria and Burkina Faso [23], limit its use in severe relapses or myasthenic crisis. Thus, in our series, the rates of use of IVIG and PE were 8% and 0%, respectively, despite the occurrence of myasthenic attacks in 22% of our patients. Similarly, in Côte d’Ivoire, none of the 5 patients had access to them due to lack of financial resources [11]. On the other hand, in Trinidad and Tobago [2, 26], 11% and 40% of patients benefited from PE and IVIG, respectively.

Numerous studies have reported substantial benefit from thymectomy in AMG [14, 41]. Its efficacy over medical therapy in AMG with or without thymic abnormalities has recently been demonstrated in several trials. These include the randomized MGTX trial conducted in 36 centers worldwide between 2006 and 2012 [45], a retrospective study using a prospective database of 3,017 patients between 1941 and 2013 in New York [20], and a systematic review of the literature published in MEDLINE up to June 2015 [42]. These studies concluded that thymectomy was superior to conservative medical treatment for the remission of AMG with or without thymic hyperplasia and expanded the indication for thymectomy to all AMG patients when possible [20]. However, the criteria for patient selection, the efficacy of thymectomy in different subtypes of AMG [32], the timing of surgery, and the optimal resection technique remain to be determined [40]. In our series, after a median follow-up of 53 months after thymectomy for thymoma or thymic hyperplasia, 44% of these patients had a stable clinical remission with discontinuation of medication or pharmacological remission with continuation of medication compared to 4% of patients who received medication alone. Our results thus confirm the superior efficacy of thymectomy over medical treatment, despite delays in its implementation.

In sub-Saharan Africa, published series report an unfavorable long-term clinical course of AMG characterized by a low rate of stable complete remission: 20% in Senegal in a pediatric series [38], 6.3% in Kenya [7], 5.5% in Trinidad and Tobago [26], and no patients in Madagascar [36], compared to 35% of all our patients, close to the 40% observed in Lithuania [35]. The most commonly reported clinical outcome was partial resolution of symptoms (clinical improvement), observed in 68.8% of patients in Kenya [7], 57.1% in Trinidad and Tobago [26] and Madagascar [36], and 60% in Senegal [38], compared with 43% of all patients in our series. Treatment refractoriness, characterized by lack of clinical improvement (or stable clinical status) despite treatment, is also quite common in SSA: 28% of patients in Madagascar [36], 10% in Senegal [38], 7% in Kenya [7] and 7% in patients in our series. Several factors explain the generally unfavorable long-term clinical course of AMG in SSA: delays in diagnosis in the severe stages of the disease due to patients’ prolonged wandering, and difficulties in diagnosis and management due to the unavailability and/or inaccessibility of paraclinical tests (autoantibodies assays) and therapies with proven efficacy for patients. In SSA, the vast majority of patients with AMG do not have access to thymectomy due to lack of adequate technical facilities or availability of thoracic surgeons [7, 36, 38]. In our series, 40% of patients still benefited from thymectomy, partly due to the availability of a technical platform and three thoracic surgeons.

In developed countries, the mortality rate of AMG has decreased significantly to less than 5% due to the availability of sophisticated diagnostics, emergency immunologic therapies with proven efficacy such as IVIG and PE, and long-term immunomodulatory therapies such as thymectomy and monoclonal antibodies, combined with appropriate resuscitation measures. Current mortality is related to the severity of respiratory impairment and treatment complications [13, 32, 35]. In China, mortality rates as low as 1.5% have recently been reported [9]. In SSA, mortality rates are still quite high, ranging from 27.5% in Nigeria [30], 16.7% in-hospital and 25% during long-term follow-up in Kenya [7], 15% in our series, 14% in Madagascar [36], and 10% in children in Senegal [38]. These high mortality rates in our context can be explained by the unavailability and/or unaffordability of treatments for the management of severe, rapidly fatal myasthenic attacks or flare-ups. The unavailability of monoclonal antibodies such as rituximab for the treatment of refractory cases and delays in diagnosis in the severe generalized phase of the disease are also risk factors for death in myasthenic patients living in SSA [22, 23, 44]. In our series, only the diagnosis of AMG in the severe generalized stage was significantly associated (p=0,02) with an unfavorable outcome, defined by the finding of a severe to very severe clinical stage (MGFA stage IV or V) or the occurrence of AMG-related death at the end of follow-up. When AMG-related mortality is considered, it is closely related to the involvement of respiratory and bulbar innervation muscles, specific to the severe generalized stage [5, 21, 46]. On the other hand, several studies have also identified late-onset AMG and the coexistence of AMG with other autoimmune diseases as major risk factors for an unfavorable outcome [3, 5, 10, 13, 21, 29, 46]. Late-onset AMG is associated with a higher rate of thymoma [5, 29]. Older patients are less likely to achieve a complete and stable remission [3] and are more likely to experience a myasthenic crisis with an unfavorable prognosis, including death [10]. Their management is also difficult due to comorbidities and a higher risk of acetylcholinesterase- or corticosteroid-related side effects [13]. In addition, several studies have identified 3 risk factors for secondary generalization of AMG: thymic hyperplasia, AMG-specific antibody se-ropositivity, and autoimmune comorbidities [46]. Other studies have shown that early and prolonged exposure of patients to immunosuppressive therapies (especially corticosteroids) is the most important protective factor against disease worsening [5, 21, 46].

Early diagnosis, improved access to and availability of diagnostic and therapeutic resources, and long-term immunotherapy (especially thymectomy, immunosuppressive drugs, and monoclonal antibodies) would help reduce mortality. Government subsidies and patient education based on patient associations would help to improve the functional prognosis and quality of life of patients followed for AMG in SSA.

## Strengths and limitations of our study

The small number of patients in our study and the lack of anti-RACh and anti-MuSK antibodies assays in a relatively large proportion of our patients due to lack of financial resources may have introduced bias.

Similarly, the hospital-based nature of our study may have introduced a selection bias toward the most severe cases at the expense of purely ocular or mild generalized clinical forms.

These limitations are largely offset by the advantages of this study: median longitudinal follow-up of 53 months (IQ=10, extremes 12 and 120 months); diagnosis of AMG based on a range of clinical, paraclinical and/or therapeutic arguments; anti-RACh or anti-MuSK antibodies assays performed in the same biomedical analysis laboratory; EMNG examinations performed on the same type of equipment; high proportion of patients who underwent thymectomy in the same center by the same surgical team.

## Conclusion

In Burkina Faso, AMG is suffering from delayed diagnosis. Almost all patients treated for AMG benefit from long-term treatment with anticholinesterase and corticosteroids alone or in combination with azathioprine. Although thymectomy is available, its availability is limited and waiting lists are long. The clinical course is often characterized by myasthenic attacks and relapses. Lethality remains high at about 1 in 10 cases. Clinical remission affects only about 1/3 of surviving patients due to the unavailability and unaffordability of treatments. Improving access to and availability of antimyasthenic drugs, particularly through government subsidies, patient education and patient associations, will make it possible to reduce morbidity and mortality and improve the functional prognosis of patients followed up for AMG in our context.

## Funding

No funding was provided for this work.

## Authors’ contributions

LOMPO Djingri Labodi: study design, literature search, data collection, study protocol development, data analysis, drafting of the manuscript. NACOULMA Hervé: data collection, data analysis, literature search.

KYELEM Julie Marie Adeline: data collection, revision and validation of the protocol, revision of the manuscript.

ZOUNGRANA Alassane: data collection, validation of the protocol.

KERE M Fabienne Yabtouta: data collection, drafting of the manuscript.

GNAMPA Melody Zeinab: data collection. NAPON Christian: protocol validation, study supervision.

MILLOGO Athanase: study protocol validation, study supervision.

## Conflicts of interest

The authors declare no conflict of interests.
